# Supplementary material for: Modeling glioblastoma heterogeneity as a dynamic network of cell states
Source: Mol Syst Biol. 2021 Sep 16;17(9):e10105. doi: 10.15252/msb.202010105 (PMC8444284; doi:10.15252/msb.202010105)
Supplement: Supplementary file 5 — Source Data for Figure 3 [file MSB-17-e10105-s001.zip › Figure3A_sourcedata/GSEA_3065/hallmarks_state5.GseaPreranked.1623416302988/HALLMARK_WNT_BETA_CATENIN_SIGNALING.html]

Details for gene set HALLMARK\_WNT\_BETA\_CATENIN\_SIGNALING[GSEA]

|  || Dataset | state5 |
| Phenotype | NoPhenotypeAvailable |
| Upregulated in class | na\_pos |
| GeneSet | HALLMARK\_WNT\_BETA\_CATENIN\_SIGNALING |
| Enrichment Score (ES) | 0.36970264 |
| Normalized Enrichment Score (NES) | 1.1418953 |
| Nominal p-value | 0.24788733 |
| FDR q-value | 0.26244152 |
| FWER p-Value | 0.897 |
Table: GSEA Results Summary

  

Fig 1: Enrichment plot: HALLMARK\_WNT\_BETA\_CATENIN\_SIGNALING      
 Profile of the Running ES Score & Positions of GeneSet Members on the Rank Ordered List

  

| PROBE | GENE SYMBOL | GENE\_TITLE | RANK IN GENE LIST | RANK METRIC SCORE | RUNNING ES | CORE ENRICHMENT || 1 | NOTCH1 |  |  | 348 | 0.361 | 0.0927 | Yes |
| 2 | ADAM17 |  |  | 711 | 0.259 | 0.1465 | Yes |
| 3 | CSNK1E |  |  | 1134 | 0.194 | 0.1699 | Yes |
| 4 | JAG1 |  |  | 1304 | 0.170 | 0.2131 | Yes |
| 5 | NCSTN |  |  | 1348 | 0.166 | 0.2689 | Yes |
| 6 | DKK1 |  |  | 1388 | 0.163 | 0.3238 | Yes |
| 7 | PTCH1 |  |  | 1526 | 0.150 | 0.3629 | Yes |
| 8 | TCF7 |  |  | 1863 | 0.122 | 0.3697 | Yes |
| 9 | PSEN2 |  |  | 2293 | 0.092 | 0.3554 | No |
| 10 | NUMB |  |  | 2641 | 0.071 | 0.3425 | No |
| 11 | MAML1 |  |  | 2795 | 0.063 | 0.3483 | No |
| 12 | CTNNB1 |  |  | 3417 | 0.031 | 0.2903 | No |
| 13 | KAT2A |  |  | 3748 | 0.018 | 0.2599 | No |
| 14 | DVL2 |  |  | 3959 | 0.009 | 0.2396 | No |
| 15 | CCND2 |  |  | 4539 | -0.016 | 0.1807 | No |
| 16 | MYC |  |  | 4656 | -0.020 | 0.1751 | No |
| 17 | GNAI1 |  |  | 4664 | -0.021 | 0.1818 | No |
| 18 | SKP2 |  |  | 5018 | -0.034 | 0.1546 | No |
| 19 | HDAC5 |  |  | 5064 | -0.035 | 0.1625 | No |
| 20 | LEF1 |  |  | 5255 | -0.042 | 0.1567 | No |
| 21 | TP53 |  |  | 5275 | -0.043 | 0.1703 | No |
| 22 | NCOR2 |  |  | 5345 | -0.047 | 0.1795 | No |
| 23 | RBPJ |  |  | 5474 | -0.051 | 0.1839 | No |
| 24 | AXIN1 |  |  | 5642 | -0.060 | 0.1870 | No |
| 25 | CUL1 |  |  | 6216 | -0.086 | 0.1543 | No |
| 26 | HEY1 |  |  | 7370 | -0.177 | 0.0901 | No |
| 27 | HDAC2 |  |  | 7865 | -0.247 | 0.1249 | No |
Table: GSEA details [plain text format]

  

Fig 2: HALLMARK\_WNT\_BETA\_CATENIN\_SIGNALING: Random ES distribution      
 Gene set null distribution of ES for **HALLMARK\_WNT\_BETA\_CATENIN\_SIGNALING**
